# HPV prevalence and type distribution in Cypriot women with cervical cytological abnormalities

**DOI:** 10.1186/s12879-017-2439-0

**Published:** 2017-05-16

**Authors:** George Krashias, Dana Koptides, Christina Christodoulou

**Affiliations:** Department of Molecular Virology, The Cyprus Institute of Neurology and Genetics, 6 International Airport Avenue, 2370 Nicosia, Cyprus

**Keywords:** Human papillomavirus (HPV), Prevalence, Typing, Age distribution, Cytology, Cyprus

## Abstract

**Background:**

Human papillomavirus (HPV) is the most common sexually transmitted agent, and it can cause cervical lesions and cancer in females. Currently, information regarding the prevalence of HPV in Cyprus is lacking. The aim of this study was to evaluate the HPV type-specific prevalence in 596 women, aged 19–65 years, with cytological abnormalities. Additionally, in a subset of 348 women for whom cytology results of the Pap test were available, the association between HPV infection and cervical disease was investigated.

**Methods:**

HPV detection and typing was carried out using PCR and restriction fragment length polymorphism analysis, respectively.

**Results:**

Overall, the HPV prevalence was 72.8%, and it was shown to be age dependent, with a decreasing prevalence until the age of 45 years (*p* = 0.0018, χ^2^). Two hundred and fifty-eight women (59.4%) were infected with high-risk HPV, 151 (34.8%) with low-risk HPV, and 25 (5.8%) with HPV types of unknown risk. The most common high-risk HPV type was HPV16 (17.7%), followed by HPV31 (12.9%), HPV58 (7.1%), HPV68 (4.6%), HPV18 (4.1%), and HPV56 (3.7%). Among the women for whom cytology results were available, 268 (77%) were HPV positive, with a sample distribution as follows: 188 (74%) had atypical squamous cells of undetermined significance (ASCUS), 61 (85.9%) had low-grade squamous intraepithelial lesion (L-SIL), and 19 (82.6%) had high-grade squamous intraepithelial lesion (H-SIL). HPV16 was the most common type among women affected by L-SIL (19.7%) and H-SIL (15.8%), with HPV31 being the most common type in women affected by ASCUS (16.5%).

**Conclusions:**

The present study provides the first epidemiological data related to HPV prevalence and type distribution in Cypriot women with cytological abnormalities.

## Background

Human papillomavirus (HPV) is the most common sexually transmitted pathogen in both men and women. Accumulating epidemiological evidence supports a strong association between HPV and genital warts as well as cancer of the cervix, vulva, vagina, anus, and penis [1–3]. Cervical cancer is the second most common cancer in women worldwide [3, 4]. It is estimated that approximately 500,000 new cases of cervical cancer are diagnosed each year, with a 56% mortality rate in the developing world [3, 4].

Currently, over 100 different HPV types have been described, and they can be further subdivided into low-risk and high-risk types according to their oncogenic potential [5]. According to the International Agency for Research on Cancer (IARC) Monograph (2012), there are 13 high-risk HPV (HR-HPV) types, including HPV16, 18, 31, 33, 35, 39, 45, 51, 52, 56, 58, 59, and 68 [3, 6, 7]. HPV16 and 18 have been associated with 70% of cervical cancer cases [6, 8, 9], with other HR-HPV types being responsible for 20% of cervical cancers globally [6, 8, 9]. Low-risk HPV (LR-HPV) types include HPV6, 11, 42, 43, and 44, which are known to be associated with hyperplastic lesions such as genital warts [5].

The oncogenic potential of particular HPV types highlights the importance of the detection and typing of different HPV isolates. The results of HPV testing have significant prognostic and therapeutic implications, providing clinicians with valuable information for deciding the most appropriate course of action for each individual patient [10]. In addition, data regarding HPV type-specific distribution can provide a valuable tool in the quest for the implementation of vaccination programmes against cervical cancer. Currently, there are three prophylactic HPV vaccines available (Gardasil, Gardasil 9, and Cervarix) that are approved for use in many countries around the world [11–13]. All three vaccines provide protection against infection with HR-HPV16 and HR-HPV18. Gardasil also includes prevention against LR-HPV6 and LR-HPV11, whereas the recently approved Gardasil 9 expands protection further by adding five additional HR-HPV types (31, 33, 45, 52, and 58) [11–13]. Until recently, Cyprus was one of the very few EU countries yet to introduce a national HPV immunization programme. In January 2016, the Health Ministry introduced an HPV vaccination programme for all 12-year-old girls.

The government of Cyprus has not yet established any form of a national health system, and thus, cervical screening in the country is performed on an ad hoc basis and can be conducted in public or private hospitals or clinics [14]. To the best of our knowledge, epidemiological studies in Cyprus regarding the distribution of HPV types in the general population or in women with cytological abnormalities are still lacking. This study aimed to provide data regarding HPV prevalence and type distribution in Cypriot women with cervical cytological abnormalities. Importantly, the data presented here could provide a valuable baseline for assessing the impact of the newly introduced vaccination programme in the future.

## Methods

### Study population

The data for the present study were gathered between October 2012 and May 2015 from the results of diagnostic HPV testing of samples from women with abnormal Pap tests that were referred by experienced gynaecologists. The study included cervicovaginal wash samples from 596 women [mean age 37.3 years, standard deviation (SD) 11.9 years, range 19–65 years] attending private or governmental gynaecological outpatient clinics in Cyprus. The samples were tested for HPV at the Molecular Virology department of the Cyprus Institute of Neurology and Genetics. Cytology results were available only for a subgroup of 348 women. Based on the Bethesda system classification [15] cervical cytological samples were classified as follows: atypical squamous cells of undetermined significance (ASCUS), low-grade squamous intraepithelial lesion (L-SIL) or high-grade squamous intraepithelial lesion (H-SIL). Age class distribution among the participating women is reported in Table 1.

**Table 1:** Results of HPV detection by age and cytology

	Outcomes of HPV detection and typing, *N*/% (95% C.I)					
	HPV-	HPV+	HR-HPV+	LR-HPV+	UR-HPV+	Total (%)
Total^a^	162/27.2% (23.8–30.9)	434/72.8% (69.1–76.2)	258/59.4% (54.8–64)	151/34.8% (30.5–39.4)	25/5.8% (3.9–8.4)	596 (100%)
Age^a^ (years)						
≤ 25	15/15.3% (9.4–23.8)	83/84.7% (76.2–90.6)	52/62.6% (51.9–72.3)	31/37.4% (27.7–48.1)	0 (0%) (<0.01–7.2)	98 (100%)
26–35	48/23.1% (17.9–29.3)	160/76.9% (70.7–82.2)	98/61.3% (53.5–68.5)	54/33.7% (26.9–41.4)	8/5% (2.4–9.7)	208 (100%)
36–45	49/36.6% (28.9–45)	85/63.4% (55–71.1)	48/56.5% (45.9–66.5)	28/32.9% (23.9–43.5)	9/10.6% (5.5–19.1)	134 (100%)
≥ 46	50/32.1% (25.2–-39.7)	106/67.9% (60.3–74.8)	60/56.6% (47.1–65.7)	38/35.8% (27.4–45.3)	8/7.6% (3.7–14.4)	156 (100%)
Cytology^b^						
ASCUS	66/26% (21–31.7)	188/74% (68.3–79)	117/62.2% (55.1–68.9)	62/33% (26.7–40)	9/4.8% (2.4–9)	254 (100%)
L-SIL	10/14.1% (7.6–24.2)	61/85.9% (75.8–92.4)	37/60.7% (48.1–72)	22/36.1% (25.2–48.6)	2/3.2% (0.3–11.9)	71 (100%)
H-SIL	4/17.4% (6.4–37.7)	19/82.6% (62.3–93.6)	11/57.9% (36.2–76.9)	7/36.8% (19.1–59.1)	1/5.3% (<0.01–26.5)	23 (100%)

^a^HPV detection and typing in 596 women

^b^HPV detection and typing in 348 women for which cytology results were available

### Extraction of DNA and detection of HPV infection by PCR

Cervicovaginal wash samples were collected by washing the cervical and vaginal wall with 10 mL of normal saline and then immediately shipped at 4 °C to the Molecular Virology department, where DNA extraction was performed the same day. Each vial was vortexed for 15 s before use. Starting material (400 μL) was then used for DNA extraction using the iPrep PureLink Virus Kit (Life Technologies, Carlsbad, CA, USA), according to manufacturer’s instructions. The resulting DNA was immediately processed for the detection and typing of HPV DNA.

A PCR assay using the MY09/MY11 L1 consensus primer set was performed as previously described [16]. Specifically, the test uses the primers MY09 (reverse: 5′ CGT CCM ARR GGA WAC TGA TC 3′) and MY11 (forward: 5′ GCM CAG GGW CAT AAY AAT GG 3′) to amplify approximately a 450 bp fragment within the L1 open reading frame in the HPV genome [16]. To provide a control for sampling and cell adequacy, extraction, and amplification, an additional primer pair targeting human beta globin was used as previously described [17]. The HPV PCR test was performed in a 60 μL reaction containing 6 μL of the isolated DNA, 1× PCR buffer, 4.5 mM MgCl_2_, 0.2 mM dNTPs, 0.5 μM of forward and reverse primers each, and 2.5 U of AmpliTaq Gold DNA polymerase (Roche Molecular Systems Inc., USA). The amplification reactions were performed using an MJR PTN-200 PCR machine (MJ Research Inc., Watertown, MA, USA) with the following conditions: initial denaturation for 10 min at 95 °C, followed by 40 cycles of 15 s at 95 °C, 20 s at 55 °C, 30 s at 72 °C, and finally at 72 °C for 5 min. The resulting PCR products were evaluated by electrophoresis on a 1.8% agarose gel stained with ethidium bromide.

### HPV genotyping by restriction fragment length polymorphism (RFLP) analysis

Typing of HPV-DNA-positive samples was performed by restriction digestion of PCR products. Each restriction reaction was performed separately in a final volume of 20 μL using 5–7 μL of crude MY09/11 PCR product, 2 μL of 10× recommended restriction buffer, and 5–10 U of the following restriction endonucleases: EcoRI, BamHI, HinfI, DraI, PstI (all from New England BioLabs, Ipswich, MA, USA), according to the manufacturer’s instructions. Reactions were incubated at 37 °C for 1 h. Digested products were electrophoretically separated on 2% agarose gels supplemented with ethidium bromide in parallel with a 100 bp DNA ladder (New England BioLabs, Ipswich, MA). In cases where the HPV types were unidentifiable due to the similarity of restriction profile, or a mixture of HPV types was detected, a second round of restriction digestion of PCR products was performed using the above protocol with the following restriction endonucleases: AccI, HaeIII, HincII, HindIII, SpeI, and SspI (all from New England BioLabs, Ipswich, MA), according to the manufacturer’s instructions.

Following the RFLP analysis, individual HPV genotypes were classified into HR, LR, and unknown risk (UR). Classification of HR-HPV genotypes followed the IARC Monograph (2012) [7] and included HPV genotypes 16, 18, 31, 33, 35, 39, 45, 51, 52, 56, 58, 59, and 68. The LR group included HPV genotypes 6, 11, 42, 44, 53, 54, 55, 61, 66, 70, 73, 81, 82, and 89. The UR group included HPV genotypes 34, 62, 67, 83, 84, 85, 90, 91, and 118.

### Statistical analysis

Overall HPV prevalence was initially determined in all 596 samples included in the study, followed by analysis to determine type-specific distribution in those samples with HPV-positive status. These results were also analysed in the context of the different age groups. The association between HPV infection and individual genotypes with the three different groups of cytology result (ASCUS, L-SIL, H-SIL) was assessed in 348 samples for which cytology results were available.

The GraphPad Prism software version 5 (GraphPad, San Diego, CA, USA) was used for statistical analysis. Fisher’s exact test was used to compare proportions where appropriate. Trends in proportion were tested using the chi-square test (χ^2^). Proportions were computed with the corresponding 95% confidence intervals (95% CI) according to the modified Wald method [18]. All statistical tests were two-sided, and all *P* values of <0.05 were considered statistically significant.

### Ethical approval

This study was exempted from ethical approval by the Cyprus National Bioethics Committee (Reference no: ΕΕΒΚ/21.1.02.01.05). Ethical approval and informed consent was not required because all the data utilized for the present study were generated following diagnostic HPV tests of cervicovaginal wash samples that were referred to the department of Molecular Virology by experienced gynaecologists as part of a diagnostic procedure in women with an abnormal Pap test.

## Results

### HPV prevalence and age distribution

Of the 596 women tested, 434 women (72.8%) were positive for at least one HPV type; the prevalence among age groups is listed in Table 1. Overall, the HPV prevalence was age dependent, with a decreasing prevalence until the age of 45 years (*p* = 0.0018, χ^2^). The highest prevalence of HPV was detected in women ≤25 years (84.7%), followed by women aged 26–35 years (76.9%), and women ≥46 years (67.9%), and the lowest prevalence of HPV infection (63.4%) was found in women between 36 and 45 years (Table 1).

### HPV genotype distribution

The 434 samples identified to be positive for HPV DNA were further analysed to identify the infecting HPV genotype. In total, 36 HPV types were identified and stratified according to their oncogenic potential into the following three categories: 13 HR-HPV types (HPV16, 18, 31, 33, 35, 39, 45, 51, 52, 56, 58, 59, and 68), 14 LR-HPV types (HPV6, 11, 42, 44, 53, 54, 55, 61, 66, 70, 73, 81, 82, and 89), and 9 types for which the risk is still unknown (UR-HPV) (HPV34, 62, 67, 83, 84, 85, 90, 91, and 118). The prevalences of HR-HPV, LR-HPV, and UR-HPV are listed in Table 1. In general, regardless of patient age, the HR-HPV types were the most frequent (59.4% of the overall HPV prevalence), with the highest prevalence (62.6%) in women ≤25 years. Although not age dependent (*p* > 0.05, χ^2^), the prevalence of HR-HPV types declined marginally as age increased up to the group of women ≥46 years of age (26–35: 61.3%; 36–45: 56.5%; ≥46: 56.6%). In contrast, the prevalence of UR-HPV appeared to be age dependent (*p* = 0.0232, χ^2^), with an increasing prevalence with increasing age up to the 36–45 age group (10.6%). No significant association could be established between the LR-HPV group and any of the age groups. The prevalence of LR-HPV types ranged from 37.4% in the youngest age group, to 33.7%, 32.9% and 35.8% in the age groups 26–35, 36–45, and ≥46 respectively (Table 1).

**Fig. 1:**
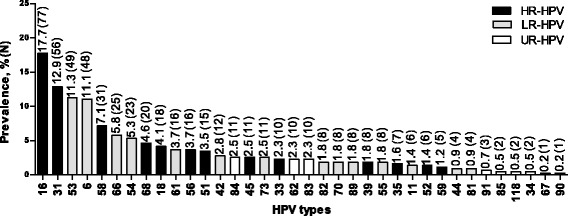
Overall HPV type-specific distribution in decreasing order of prevalence among HPV-positive women (*N* = 434)

Detailed analysis of the prevalence of individual HPV genotypes in the whole cohort of samples revealed that HR-HPV16 was the most common type detected (17.7%), followed by HR-HPV31 (12.9%) (Fig. 1). The remaining HR-HPV types detected in order of decreasing prevalence were HPV58 (7.1%), HPV68 (4.6%), HPV18 (4.1%), HPV56 (3.7%), HPV51 (3.5%), HPV45 (2.5%), HPV33 (2.3%), HPV39 (1.8%), HPV35 (1.6%), HPV52 (1.4%), and HPV59 (1.2%) (Fig. 1). Overall, the frequencies of infections with HPV types of LR and UR were lower than the occurrence of HR-HPV infections, with the most common LR- and UR-HPV types being HPV53 (11.3%) and HPV84 (2.5%) respectively (Fig. 1).

### Multiple infections

Co-infection with two or more HPV types was observed in 99 of the 434 HPV-positive samples (22.8%). Dual infections accounted for 18.7% of all positive samples, with three and four viruses detected in 3.9% and 0.2% of positive samples, respectively. Table 2 summarizes the prevalence of each HPV type in single vs. multiple infections. Overall, HPV16 was the most common genotype detected in multiple infections (5.8%), followed by HPV31 (4.8%), HPV53 (4.6%), HPV6 and HPV58 (2.8%), and HPV66 (2.1%). HPV types that were not present in conjunction with other viruses included HPV67, HPV85, HPV90, and HPV118, which were each present in only 1 or 2 samples.

**Table 2:** Distribution of infections in 434 HPV-positive women, by HPV genotypes detected by PCR-RFLP

Genotypes	Infections	
	Single, *N* (%)	Multiple, *N* (%)
HR-HPV		
HPV16	52 (12%)	25 (5.8%)
HPV18	11 (2.5%)	7 (1.6%)
HPV31	35 (8.1%)	21 (4.8%)
HPV33	4 (0.9%)	6 (1.4%)
HPV35	5 (1.2%)	2 (0.5%)
HPV39	5 (1.2%)	3 (0.7%)
HPV45	6 (1.4%)	5 (1.2%)
HPV51	10 (2.3%)	5 (1.2%)
HPV52	1 (0.2%)	5 (1.2%)
HPV56	8 (1.8%)	8 (1.8%)
HPV58	19 (4.4%)	12 (2.8%)
HPV59	3 (0.7%)	2 (0.5%)
HPV68	14 (3.2%)	6 (1.4%)
LR-HPV		
HPV6	36 (8.3%)	12 (2.8%)
HPV11	3 (0.7%)	3 (0.7%)
HPV42	11 (2.5%)	1 (0.2%)
HPV44	2 (0.5%)	2 (0.5%)
HPV53	29 (6.7%)	20 (4.6%)
HPV54	17 (3.9%)	6 (1.4%)
HPV55	4 (0.9%)	4 (0.9%)
HPV61	10 (2.3%)	6 (1.4%)
HPV66	16 (3.7%)	9 (2.1%)
HPV70	6 (1.4%)	2 (0.5%)
HPV73	4 (0.9%)	7 (1.6%)
HPV81	3 (0.7%)	1 (0.2%)
HPV82	5 (1.2%)	3 (0.7%)
HPV89	6 (1.4)	2 (0.5%)
UR-HPV		
HPV34	1 (0.2%)	1 (0.2%)
HPV62	5 (1.2%)	5 (1.2%)
HPV67	1 (0.2%)	0 (0%)
HPV83	5 (1.2%)	5 (1.2%)
HPV84	6 (1.4%)	5 (1.2%)
HPV85	2 (0.5%)	0 (0.0%)
HPV90	1 (0.2%)	0 (0.0%)
HPV91	2 (0.5%)	1 (0.2%)
HPV118	2 (0.5%)	0 (0.0%)

### Prevalence of HPV types stratified by the cervical cytological result

In the subgroup of 348 women for whom a cytology result was available, 254 (73%) were affected by ASCUS, 71 (20.4%) by L-SIL and 23 (6.6%) by H-SIL (Table 1). HPV DNA was detected in 268 out of 348 of these samples (77%). Among women with a cytology result of ASCUS, L-SIL and H-SIL, 74%, 85.9% and 82.6% were infected with HPV, respectively (Table 1).

The prevalence of HR-HPV, LR-HPV and UR-HPV types among HPV-positive samples diagnosed with ASCUS, L-SIL and H-SIL is also listed in Table 1. Irrespective of cytology results, HR-HPV genotypes were detected with the highest prevalence in all three cytology classes (ASCUS: 62.2%, L-SIL: 60.7%, H-SIL: 57.9%). The second most prevalent HPV genotypes that were detected belonged to the LR-HPV group, with their prevalence ranging from 33% in the ASCUS group to 36.1% in the L-SIL group and 36.8% in the H-SIL group. No significant association could be established between the different classes of cytological abnormalities and any of the HR-HPV, LR-HPV, and UR-HPV type groups (*p* > 0.05, χ^2^).

Detailed analysis of the prevalence of individual HPV genotypes in the three different classes of cytological abnormalities revealed that the most prevalent HPV types were: HPV31 in 31 out of 188 HPV-positive women affected by ASCUS (16.5%), HPV16 in 12 out of 61 HPV-positive women affected by L-SIL (19.7%) and HPV16 in 3 out of 19 HPV-positive women affected by H-SIL (15.8%). The distribution and proportion of the HPV genotypes in different classes of cytological abnormalities are presented in Table 3.

**Table 3:** Distribution and proportion of HPV genotypes in ASCUS, L-SIL, and H-SIL cytological lesions

	Cytology result, *N* (%)			
Genotypes	ASCUS (*N* = 188)	L-SIL (*N* = 61)	H-SIL (*N* = 19)	Total^a^ (%)
HR-HPV				
HPV16	26 (13.8%)	12 (19.7%)	3 (15.8%)	41 (15.3%)
HPV18	8 (4.3%)	2 (3.3%)	1 (5.3%)	11 (4.1%)
HPV31	31 (16.5%)	7 (11.5%)	2 (10.5%)	40 (14.9%)
HPV33	4 (2.1%)	2 (3.3%)	0 (0%)	6 (2.2%)
HPV35	3 (1.6%)	1 (1.6%)	0 (0%)	4 (1.5%)
HPV39	5 (2.7%)	2 (3.3%)	0 (0%)	7 (2.6%)
HPV45	5 (2.7%)	1 (1.6%)	2 (10.5%)	8 (3%)
HPV51	11 (5.9%)	1 (1.6%)	1 (5.3%)	13 (4.9%)
HPV52	0 (0%)	2 (3.3%)	2 (10.5%)	4 (1.5%)
HPV56	3 (1.6%)	6 (9.8%)	0 (0%)	9 (3.4%)
HPV58	18 (9.6%)	2 (3.3%)	0 (0%)	20 (7.5%)
HPV59	3 (1.6%)	1 (1.6%)	0 (0%)	4 (1.5%)
HPV68	8 (4.3%)	5 (8.2%)	1 (5.3%)	14 (5.2%)
LR-HPV				
HPV6	15 (8%)	7 (11.5%)	2 (10.5%)	24 (9%)
HPV11	2 (1.1%)	1 (1.6%)	0 (0%)	3 (1.1%)
HPV42	9 (4.8%)	1 (1.6%)	0 (0%)	10 (3.7%)
HPV44	1 (0.5%)	1 (1.6%)	1 (5.3%)	3 (1.1%)
HPV53	21 (11.2%)	5 (8.2%)	1 (5.3%)	27 (10.1%)
HPV54	6 (3.2%)	1 (1.6%)	2 (10.5%)	9 (3.4%)
HPV55	4 (2.1%)	0 (0%)	1 (5.3%)	5 (1.9%)
HPV61	11 (5.9%)	3 (4.9%)	0 (0%)	14 (5.2%)
HPV66	8 (4.3%)	7 (11.5%)	0 (0%)	15 (5.6%)
HPV70	2 (1.1%)	1 (1.6%)	0 (0%)	3 (1.1%)
HPV73	2 (1.1%)	3 (4.9%)	0 (0%)	5 (1.9%)
HPV81	3 (1.6%)	0 (0%)	0 (0%)	3 (1.1%)
HPV82	6 (3.2%)	0 (0%)	0 (0%)	6 (2.2%)
HPV89	4 (2.1%)	1 (1.6%)	1 (5.3%)	6 (2.2%)
UR-HPV				
HPV34	0 (0%)	1 (1.6%)	0 (0%)	1 (0.4%)
HPV62	1 (0.5%)	3 (4.9%)	0 (0%)	4 (1.5%)
HPV83	5 (2.7%)	0 (0%)	1 (5.3%)	6 (2.2%)
HPV84	5 (2.7%)	1 (1.6%)	2 (10.5%)	8 (3%)
HPV90	0 (0%)	1 (1.6%)	0 (0%)	1 (0.4%)
HPV91	1 (0.5%)	0 (0%)	0 (0%)	1 (0.4%)

^a^Overall prevalence of individual HPV types in 268 HPV-positive women with available cytology result

## Discussion

The analysis presented here is a study of the rate of HPV detection and type distribution in samples of women living in Cyprus that were referred because of an abnormal Pap test. To the best of our knowledge, this work is the first of its kind in this population.

Overall, the prevalence of HPV in the entire group (*n* = 596) was 72.8% (434 of 596). This result is consistent with previous studies of similar nature conducted in other European countries that reported HPV prevalence ranging from 35.3% to 88.9% among women with cytological abnormalities [19–27]. The results for each of these studies together with the methods used for analysis are listed in Table 4. The high HPV prevalence in our study group could be partly attributed to the fact that the referred samples were selected for molecular investigation by experienced specialists based on cytological abnormalities and therefore were more likely to have detectable HPV DNA as a result of a current HPV infection. Variations between studies most likely reflect differences in the population studied with respect to risk factors for exposure to HPV and methods used for analysis as well as triage for HPV testing by gynaecologists.

**Table 4:** Summary of European studies regarding the prevalence of HPV in women with cytological abnormalities

Authors (Year)	Citation	*N* (Country)	Method of Analysis	HPV+ (%)
Bonde et al. (2014)	[7]	5068 (Denmark)	CLART	88.9%
Capra et al. (2008)	[19]	970 (Italy)	Nested PCR/sequencing INNO-LiPA	37.7%
Delgado et al. (2012)	[20]	106 (Spain)	Linear Array	69.8%
Meloni et al. (2014)	[21]	650 (Italy)	INNO-LiPA	52.6%
Monsonego et al. (2008)	[22]	575 (France)	Linear Array Hybrid Capture II	88.1%
Panotopoulou et al. (2007)	[23]	997 (Greece)	PCR/sequencing	75.1%
Rassu et al. (2005)	[24]	1335 (Italy)	PCR-RFLP	35.3%
Simanaviciene et al. (2015)	[25]	547 (Lithuania)	PCR	67.6%
Spinillo et al. (2009)	[26]	1218 (Italy)	INNO-LiPA	69.9%
Yapijakis et al. (2008)	[27]	263 (Greece)	PCR-RFLP	81.7%

HPV prevalence stratified by age revealed an age-dependent association, in agreement with data reported in other European countries [21, 28, 29], reflecting a higher risk in young sexually active women that tend to have multiple partners [30].

In the present study, a spectrum of 36 genotypes were identified, with HR-HPV types being more frequently detected than LR-HPV types. Thirteen HR-HPV genotypes were detected in the sample cohort and accounted for 59.4% of HPV-positive samples, in accordance with other studies carried out in similar populations [19, 21, 27, 31]. Overall, the most prevalent genotype was HPV16, present in 17.7% of the specimens, followed by HR-HPV31, LR-HPV53, and LR-HPV6. These findings paralleled those of other studies, in which HPV16 and HPV31 were the predominant genotypes detected in women of European origin with cervical cytological abnormalities [19, 27, 31–33]. HPV16 together with HPV18 are known to be responsible for approximately 70% of the cervical cancer cases worldwide [6, 8, 9]. The prevalence of HPV18 has been reported in other studies, ranging between 1.8% and 16.3% [20, 21, 23, 27]. In agreement with the aforementioned studies, the prevalence of HPV18 in the present study was 4.1%.

Our study showed that at least 22.8% of women were infected with two or more HPV genotypes, in accordance with other studies reporting a prevalence of multiple infections in 11% to 50% of cases [21, 23, 34]. The clinical significance of co-infection with multiple HPV genotypes remains uncertain, with some reports showing that the clearance rate in immunocompetent women is not dependent on the number of genotypes involved in the infection [35]. Additionally, it is still not clear whether co-infection with multiple HPV genotypes increases the risk of progression to cancer [36, 37].

An increasing trend in the overall prevalence of HPV infection was observed in parallel with an increase in the degree of cervical cytological abnormalities (ASCUS: 74%, L-SIL: 85.9%, H-SIL: 82.6%). In agreement with our results showing HPV positivity in 82.6% of H-SIL cases, a meta-analysis conducted using data from 423 studies revealed an overall HPV prevalence in 84% and 85% of H-SIL cases in Europe and globally, respectively [38]. The prevalence of HR-HPV genotypes has been reported to increase with the grade of cytological lesions in some [28, 31, 39] but not all published studies [20, 21, 40]. In the present work, such an association was not observed, probably due to the low number of HPV-positive L-SIL and H-SIL cases that were included for analysis. An alternative explanation might be the lack of histological results as verification for the cytology results. As previously reported, almost 40% of women with an ASCUS diagnosis could be histologically confirmed as high-grade cervical neoplasia [41]. Nevertheless, HR-HPV types were more frequently detected than LR-HPV types in all three classes of precancerous lesions (ASCUS: 62.2% vs 33%, L-SIL: 60.7% vs 36.1%, H-SIL: 57.9% vs 36.8%), highlighting the need for more frequent follow-up examinations in women who are HR-HPV-positive.

Among individual HPV types, HPV16 was the most frequently identified type in both the L-SIL (19.7%) and the H-SIL (15.8%) group, confirming that HPV16 is the most frequent HPV type associated with high-grade lesions, as was previously reported [32, 33]. The current literature suggests that HPV16 and HPV18 are more likely to progress to cervical cancer than L-SIL and H-SIL with other HPV genotypes [32, 33]. Therefore, it could be of potential benefit to the patient if HPV16- and/or HPV18-infected SIL cases are separated from those infected with other HR-HPV types for closer monitoring and surveillance. Regarding the prevalence of HPV18 in the present study, this HPV type was detected at a low prevalence in all three classes of cytological abnormalities, with the highest prevalence detected in the H-SIL group (5.3%) followed by the ASCUS group (4.3%) and the L-SIL group (3.3%). A meta-analysis carried out with data from 55 studies of women showing a prevalence of HPV18 in L-SIL and H-SIL cases of 8.6% and 7% respectively [32, 33]. However, the results obtained for HPV18 should be interpreted cautiously because, as previously indicated, the number of L-SIL and H-SIL cases available for analysis in the present study was small. Nonetheless, it is interesting to note that the short time of progression to invasive cancer as a result of infection by HR-HPV types such as HPV18, with or without transition through the pre-invasive cases, might be an additional contributor to the low prevalence of HPV18 in L-SIL and H-SIL cases in our study.

Only few studies have addressed the prevalence of HPV in women with abnormal cytology in the geographical region in Eastern Mediterranean and Middle East. In Turkey, three studies showed an overall HPV prevalence ranging from 13.7% to 52% in women with abnormal cytology [42–44], with HR-HPV types detected in 27% and 20% of L-SIL and H-SIL cases [42]. In contrast, other studies in the surrounding region reported higher HPV prevalence rates in women with abnormal cytology, such as 84% in Jordan [45] and 100% in Egypt [46]. In the Jordanian study, HR-HPV prevalence was 72.2% and 78.6% of L-SIL and H-SIL cases, respectively [45]. In agreement with our results, HPV16 was the most common genotype detected in the above studies [42–46]. In terms of overall HPV positivity (72.8%) and HR-HPV prevalence in L-SIL (60.7%) and H-SIL (57.9%) cases, our results are discordant with the above studies. These discrepancies might be attributed to the differences in study populations and designs as well as due to the different methodologies used for sample analysis.

The current results should be evaluated in the context of the strengths and limitations of the study. A major strength of this study is the fact that it provides the first estimates of the prevalence of HPV and its type-specific distribution among women from Cyprus diagnosed with cytological abnormalities by Pap test. The presented study, however, is not without limitations. First, the results relied on samples referred to our laboratory for diagnostic purposes as convenience samples. Thus, these data cannot be considered nationally representative because the Cypriot women included in the study may not be a representative sample of all the Cypriot women living in the country. Another potential limitation is the extent of missing cytological data (41.6%) that required implementation of an amputation scheme during assessment of overall HPV prevalence and type-specific distribution in the groups with different cytological abnormalities. However, our analyses suggest that there was no selection bias when assessing HPV prevalence in only those samples for which cytology results were available, as overall HPV positivity in samples with cytology results as presented in the study (*n* = 348) (77%, 95% CI: 72.3–81.1) is very similar to HPV positivity of all analysed samples (*n* = 596) (72.8%, 95% CI: 69.1–76.2). In a similar manner, type-specific distribution in HPV-positive samples with available cytology results is comparable to type-specific distribution observed in all samples with HPV-positive status (Table 3 and Fig. 1). The present study analysed samples using an established PCR-RFLP methodology that was optimized and validated in our laboratory. Although an unassuming method of a seemingly bygone era, PCR-RFLP is a robust approach for detection and genotyping of HPV and shows excellent discriminatory power to differentiate the HPV in HR or LR groups and to identify single or multiple infections [47]. Current literature suggests additional HPV assays that are commercially available for HPV testing including Hybrid Capture 2, Linear Array, INNO-LiPa, CLART, PapilloCheck, Abbott real-time PCR, and COBAS 4800 HPV test [47]. From a screening perspective, it might therefore be interesting to show the robustness of our methodology compared to the above commercially available assays in future studies.

Beginning in January 2016, the Health Ministry of the Cyprus government has introduced an HPV vaccination programme for all 12-year-old girls. The use of genotyping assays could prove pivotal for monitoring the effect of this HPV vaccination programme. The results of the present study could also provide a baseline reference for a later comparison of HPV prevalence among vaccinated women. Additionally, the data on type-specific HPV distribution presented herein can provide a reference point for evaluating the efficacy of available vaccines in conferring cross-protection against non-vaccine types, monitoring at the same time undesirable phenomena such as HPV type replacement.

## Conclusions

In conclusion, this study presents the first investigation into the prevalence of HPV infection and HPV genotype distribution in Cypriot women with abnormal cytological tests. Whether the presented results may reflect the HPV prevalence and genotype distribution in the general population remains to be seen in additional population-based epidemiological studies. Nevertheless, the high prevalence of HPV16 and HPV6 detected in the present study highlights the potential benefits of the introduction of a national HPV immunization programme. Furthermore, as seen in the present study, the circulation of HPV genotypes other than those targeted by the available HPV vaccines [11, 12] highlights the necessity of sustaining such screening programmes in the near future. Otherwise, it is possible that the available vaccines could cause a genotype redistribution with an increase in the number of women infected with HPV types other than those targeted by vaccines.

## Abbreviations

ASCUS: atypical squamous cells of undetermined significance
HPV: human papillomavirus
HR: high-risk
H-SIL: high-grade squamous intraepithelial lesion
LR: low-risk
L-SIL: low-grade squamous intraepithelial lesion
RFLP: restriction fragment length polymorphism
UR: unknown risk
